# Determinant of women's autonomy in sexual and reproductive health decision-making in Tanzania: a multilevel analysis of 2022 demographic and health survey

**DOI:** 10.3389/frph.2025.1714846

**Published:** 2026-01-13

**Authors:** Mtoro J. Mtoro, Elihuruma Eliufoo Stephano

**Affiliations:** 1 TILAM International, Dar es Salaam, Tanzania; 2 Department of Clinical Nursing, School of Nursing and Public Health, The University of Dodoma, Dodoma, Tanzania; 3 Clinical Nursing Teaching and Research Section, The Second Xiangya Hospital, Central South University, Changsha, Hunan, China

**Keywords:** decision-making, health survey, multilevel analysis, sexual and reproductive health, Tanzania women's autonomy

## Abstract

**Background:**

The critical importance of women's autonomy in sexual and reproductive health (SRH) decision-making overall well-being is widely recognized. Existing research often provides only a generic understanding without specifically identifying the nuanced socio-economic, cultural, and demographic factors that truly enable or hinder women's autonomous SRH in Tanzania. High adolescent fertility and ongoing disparities in healthcare decision-making autonomy highlight a significant gap between policy intentions and lived realities. Therefore, this study aimed to close this gap by assessing the determinants of women's autonomy in sexual and reproductive health decision-making.

**Methods:**

This study employed an analytical cross-sectional design, utilizing secondary data from the 2022 Tanzania Demographics and Health Survey. Women's autonomy in SRH decision-making was an outcome variable derived from three questions assessing autonomy over: (1) sexual relations, (2) contraceptive use, and (3) reproductive health care. This study's analysis included 9,252 women in sexual unions. The data were analyzed using multilevel mixed-effects logistic regression, employing four models to identify determinants of women's autonomy in SRH.

**Results:**

The prevalence of women's autonomy in SRH was 49.6% (95%CI: 47.5–51.8). The individual factors influencing SRH decision making autonomy were, women aged 35–49 years (AOR = 1.33, 95%CI: 1.09–1.61), women with primary education (AOR = 1.49, 95%CI: 1.31–1.70) and secondary or higher education (AOR = 2.16, 95%CI: 1.83–2.55) were more likely to have autonomy in SRH decision making. Women in rich households (AOR = 1.19, 95%CI: 1.02–1.40) and those with media exposure (AOR = 1.49, 95%CI: 1.33–1.67) had higher odds of autonomy in SRH than their counterparts. At the community level, women in rural settings (AOR = 0.73, 95%CI: 0.61-0.87) were less likely to have autonomy in SRH compared to women in urban settings.

**Conclusion:**

This study found that nearly half of Tanzanian women lacked autonomy in SRH decision-making, significantly influenced by factors such as age, education, media exposure, current employment status, parity, wealth index, and geographic region. To address these disparities, comprehensive and context-specific interventions are necessary to overcome the cultural and socio-economic challenges faced by underserved populations.

## Background

Women's autonomy in sexual and reproductive health (SRH) decision-making is a critical factor for their overall health and well-being, particularly in sub-Saharan Africa (SSA) (1). This autonomy is also fundamental to achieving Sustainable Development Goals (SDGs) 3 and 5. These goals aim to ensure healthy lives and promote well-being, while also achieving gender equality and empowering all women and girls (1–4). Despite being essential, women's autonomy in SRH decision-making remains limited in many low- and middle-income countries, including Tanzania (5). In Tanzania, the prevalence of health decision-making autonomy was estimated to be only 19% in 2015 (6), which increased to 21% in 2022 (5), highlighting a significant challenge.

The women's autonomy in SRH decision-making has substantial implications for health and welfare (7). Studies in SSA have revealed that a low proportion of women can ask their partners to use a condom or refuse sex, with rates as low as 16.6% and 18.3% respectively, in Mali, compared to higher rates in Namibia (93.4% and 92.4%) (7, 8). In Tanzania, addressing women's autonomy is crucial given the high adolescent fertility rate, which stands at 112 per 1,000, and a teenage pregnancy rate of 22% (9). The low uptake of modern contraceptives among adolescents in Tanzania, at only 15.2% (10), further exacerbates these issues, despite 96% of adolescents being aware of modern contraceptives (8, 10, 11).

Several efforts have been taken by Tanzanian government and various stakeholders to improve women's autonomy in SRH decision-making (8). The Ministry of Health in Mainland Tanzania strategized a 5-year Implementation Plan for Family Planning (2018–2022) to increase the uptake of all family planning methods (8). This plan includes expanding the availability of modern contraceptive methods at all levels of the health system. This plan aimed to expand access to at least three modern contraceptive methods at primary levels (health dispensaries and health centers) and five at secondary (regional and district hospitals) and tertiary levels (zonal referral hospitals, national hospitals, and specialized hospitals), and scaling up youth-friendly reproductive health services (8). Mobile phone-based interventions have also shown positive progress in connecting women to SRH information and services hence facilitate their autonomy (8, 11). However, several factors, including a lack of autonomy (5), are still limiting the uptake of these SRH services.

Efforts to improve women's autonomy and empower them to manage their SRH should target specific socio-demographic factors (7). Research indicates that women from rural areas, those with no education, Muslim women, women who are not working, and women whose partners have no education are less likely to make decisions regarding their reproductive health (5, 7). Age, level of education, religion, occupation, and partner's education are all associated with women's decision-making on sexual intercourse, condom use, and reproductive health (1, 7). Studies in Tanzania have also found that women's age, education level, household wealth index, and living in certain geographic zones are independently associated with higher odds of complete autonomy in healthcare decisions. In contrast, rural residence is associated with decreased odds (5). These factors focused on general healthcare decision-making, limiting the understanding of the SRH autonomy and the influencing factors.

Despite global and national commitments to enhance women's autonomy in SRH decision-making, the burden of adolescent pregnancies persists in Tanzania, hindering the achievement of SDG target 3.7 (9). SDG 3.7 calls for universal access to SRH care services, including family planning, information, and education, and the integration of reproductive health into national strategies and programs by 2030 (4). The low prevalence of modern contraceptive use and disparities in access continue to pose significant challenges to Tanzania's progress towards this goal (10). High adolescent fertility and persistent inequalities in healthcare decision-making autonomy highlight a critical gap between policy intentions and lived realities (12). While some studies have explored individual-level factors (5, 6), a comprehensive multilevel analysis is lacking, which is crucial for identifying the complex interplay of individual, household, and community-level determinants that influence women's ability to make autonomous SRH choices. This study utilized the Connell's Theory of Gender and Power (13) to understand the gender hierarchies in SRH decision-making (14). This theory put an emphasis on how gender relations operate simultaneously at micro (individual/household) and macro (community/structural) levels hence this study adopted a multilevel analysis to understand these factors (15). Therefore, understanding the multilevel determinants of women's autonomy in SRH decision-making through comprehensive analysis is essential for developing effective policies and interventions. This gap in understanding necessitates further investigation to inform targeted and effective interventions that truly empower women and accelerate progress towards the SRH-related SDGs.

## Methods

### Conceptual framework

This study has adopted Connell's theory of gender and power (13) to assess how this gender hierarchies across individual and community levels influences women's autonomy in SRH decision-making. Raewyn Connell's theory of gender and power offers a nuanced understanding of gender as a dynamic social structure, moving beyond static biological or psychological explanations. Based on Connell's theory of gender and power, this framework illustrates how the three core structures, sexual division of labor, power, and structure of cathexis operate to maintain gender hierarchies (13).

The key theoretical alignments fall under the three main constructs of the theory. The first construct is sexual division of labor. In our secondary analysis this construct encompasses variables related to economic participation and resource allocation. These variables were women's education, working status, wealth index, and distance to health facilities. These factors determine women's economic independence and bargaining power. Another construct of Connell's theory is sexual division of power. This construct captures authority and control dynamics. This construct is related to the following variables sex of household head, educational levels (both woman's and partner's), age disparities, and media exposure. These variables reflect power imbalances in decision-making. The last construct is the structure of cathexis which includes variables related to social norms and intimate relationships. According to our secondary analysis this construct related to parity, age differences between partners, and community-level factors that reflect prevailing gender expectations and emotional dynamics.

**Figure 1:**
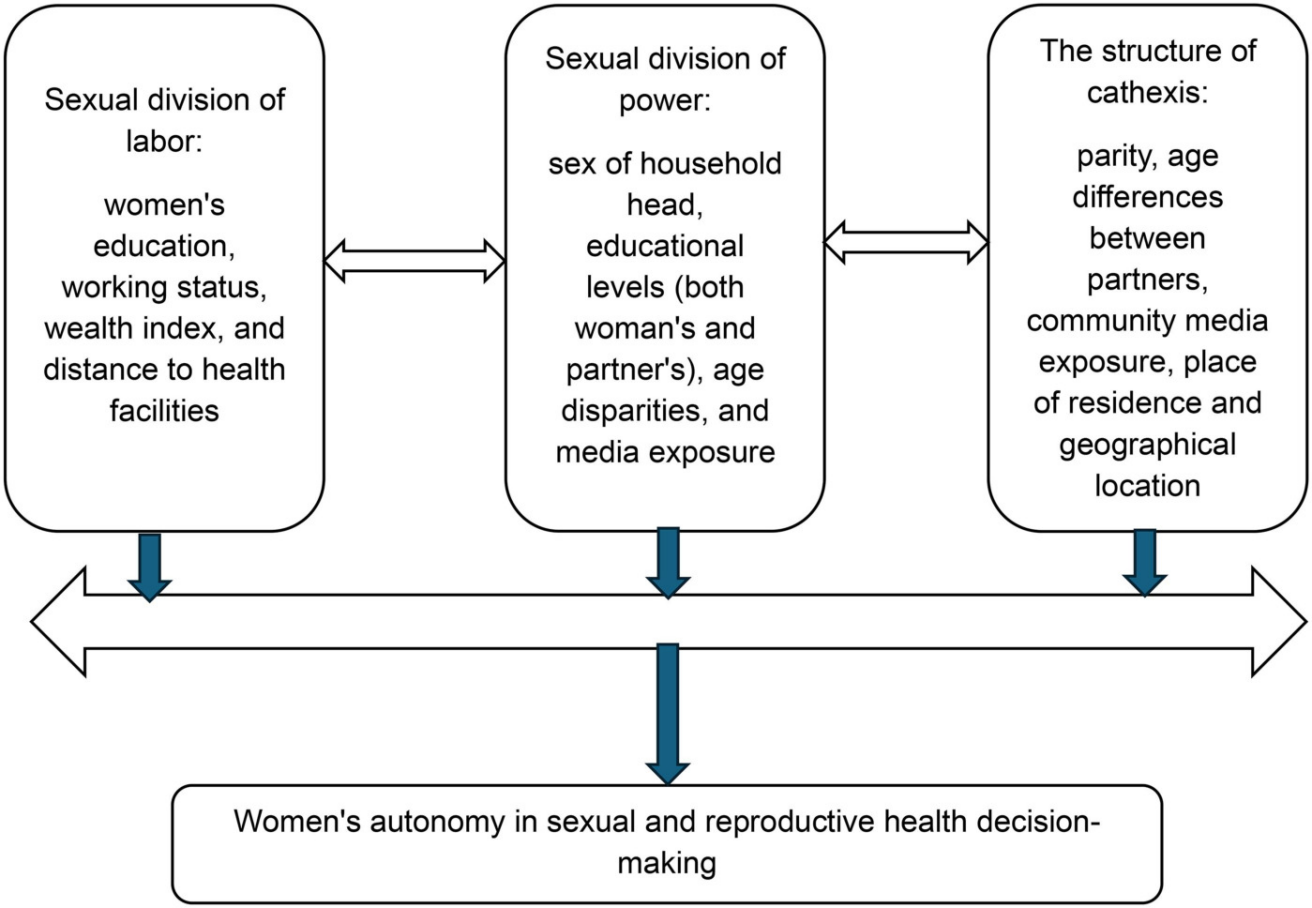
The adopted conceptual framework from the theory of gender and power, linking individual- and community-level factors influencing women autonomy in sexual and reproductive health decision-making.

The sexual division of labor, operationalized through individual factors like women's education, working status, wealth index, and distance to health facilities, directly influences economic dependency, a key mediating pathway. These individual-level resources determine a woman's bargaining power and access to services. The sexual division of power, encompassing individual factors such as the sex of the household head, educational levels, age disparities, and media exposure, directly impacts information access and social networks, which are crucial mediating pathways. These elements shape a woman's ability to participate in decision-making and access diverse perspectives. Finally, the structure of cathexis, represented by individual factors like parity and age differences between partners, alongside community-level norms, influences emotional dynamics and prevailing gender expectations. These community-level factors directly interact with a woman's individual experiences and social networks, further shaping her autonomy. Therefore, this framework helps us to understand how some of the individual-level factors combined with community-level factors through mediating pathways of economic dependency, information access, and social networks, ultimately determine women's autonomy in sexual and reproductive health decision-making see Figure 1.

### Study design and setting

An analytical cross-sectional study was conducted using secondary data from the recent Tanzania Demographic and Health Survey (TDHS). Tanzania, among the countries in East Africa, spans an area of 947,403 km^2^ with approximately 62 million people, with slightly over half being women, according to the 2022 national population census (16).

### Study sampling

The survey uses a two-stage sampling stratified sampling strategy. Firstly, clusters (Primary Sampling Units) are stratified from urban/rural settings within the geographic zones based on the most recent national population census. Secondly, households are selected from sampled clusters using equal probability sampling. The detailed TDHS methodology that includes sampling design, data collection procedures is explained elsewhere (17).

### Study population

The 2022 TDHS was conducted between February 24 and July 21, 2022, across all regions in the United Republic of Tanzania. In TDHS, women of reproductive age (WRA) (15–49 years), men, children, and households are the target population. However, this study focuses explicitly on WRA in sexual unions. This study uses Individual file records with 15,254 WRA. For the accurate measurement of women's autonomy in SRH, we excluded women who are not currently in sexual unions or never in union, and our final weighted sample was 9,252.

### Variable measurements

#### Dependent variable

The outcome variable for this study was women's autonomy in SRH decision-making, measured among women in sexual unions. It was a composite binary variable derived from three questions assessing autonomy over: (1) sexual relations, (2) contraceptive use, and (3) reproductive health care. The specific questions were: (1) ‘Can you refuse sex when you do not want to?’ (responses: yes, no, don't know/not sure); (2) ‘Who usually makes decisions about your health care?’ (responses: respondent alone, jointly with partner, partner alone, someone else, other); and (3) ‘Who makes the decision about using contraception?’ (responses: respondent alone, jointly with partner, partner alone, someone else, other). A woman was considered as having sexual autonomy in SRH (coded = 1) if she reported being able to refuse sex (response: yes), made decisions alone or jointly regarding health care, and made decisions alone or jointly regarding contraceptive use. All other combinations were considered as having no autonomy (coded = 0) (18, 19).

#### Independent variables

This study utilized variables that were accessible through TDHS and cited in the relevant literature (18–22). For comprehensive analysis, we included individual and community-level variables as stipulated in Table 1.

**Table 1:** Sociodemographic characteristics used in this study.

Variable description	Coding categories
Individual-level	
Women's age group in years	1 = 15–24, 25–34, or 35–49
Education level	1 = No formal education, 2 = Primary, or 3 = Secondary/Higher
Household wealth index	1 = Poor, 2 = Middle or 3 = Rich
Media exposure: media exposure was calculated by aggregating TV watching, radio listening, and reading newspapers and woman who has exposure to either of the media sources was categorized as having media exposure and the rest considered as having no media exposure.	1 = Yes or 2 = No
Working status	1 = Working or 2 = Not working
Parity	1 = None, 2 = 1–2 or 3 = 3+
Sex of household head	1 = Male or 2 = Female
Distance to the health facility	1 = Big problem or 2 = Not big problem
Husband/partner's age group in years	1 = 15–24, 2 = 35–34, 3 = 35–44, or 4 = 45+
Husband/partner's education	1 = No formal education, 2 = Primary or 3 = Secondary/Higher
Community-level	
Community media exposure level: The level of media exposure in the community was determined by aggregating media exposure score. It was classified as low (communities where <50% of women had media exposure) or high (communities where ≥50% of women had media exposure).	1 = Low or 2 = High
Place of residence	1 = Urban or 2 = Rural
Geographical zones	1 = Western, 2 = Northern, 3 = Central, 4 = Southern, 5 = Lake, 6 = Eastern or 7 = Zanzibar

### Descriptive statistical analysis

Descriptive statistics were used to estimate the mean (standard deviation) for continuous variables and frequency with percentages for categorical variables. Bivariate analysis using a chi-squared test of independence was used to evaluate the difference in women's autonomy in SRH across sociodemographic characteristics.

### Multilevel analysis

We employed multilevel mixed-effects logistic regression considering the hierarchical nature of the TDHS (where women are nested within households, and households are nested within clusters). Both fixed and random effects were estimated. The fixed effects (measures of association) showed the results of the association between dependent variables and the outcome variable (women's autonomy in SRH). They were reported as adjusted odds ratios (AOR) with their 95% confidence intervals (CI). Random effects (measures of variation) such as Intra-class Correlation Coefficient (ICC), Median Odds Ratio (MOR), and Proportion change in variance (PCV) were computed to measure the variation of women's autonomy in SRH across clusters (23–25). Multilevel mixed-effects logistic regression consisted of four models: Model 0 (Null model), Model I (Individual factors only), Model II (Community factors only), and Model III (Both individual and community factors). Stepwise backward selection with *p*-value <0.1 was used to select variables for inclusion in the multivariable analyses. The “melogit” package in Stata was used in fitting these models. Both Akaike's Information Criterion (AIC) and Bayesian Information Criteria (BIC) were used to measure how well the different models fitted the data. Deviance = −2 (log likelihood ratio) was used to compare the models due to the nested nature of the model; the model with the lowest deviance was selected as the best-fit model. A variance inflation factor (VIF) was used to assess for multicollinearity between independent variables before fitting a multivariable regression model. The mean VIF was <10, indicating no significant multicollinearity. All analyses were two-tailed, and a statistically significant *p*-value was set at *p*-value < 0.05.

To account for the complexity of the survey design, this study applied individual sampling weights (v005/1,000,000), primary sampling units (clusters; v021), and strata (v023) to adjust for the cluster sampling design and control sampling biases. All analyses were performed using STATA version 18 (STATA Corp, College Station, TX).

## Results

### Sociodemographic characteristics

Table 2 details sociodemographic characteristics and women's autonomy in SRH. Out of 9,252 women in sexual unions, 38.6% were aged 35–49, with a mean age of 32.8 (±8.7). Over half (58.4%) had primary education, and 43.9% belonged to wealthier households. Two-thirds (66.3%) had media exposure, and 68.1% were employed. Most households (86.1%) were male-headed, and 68.4% reported that distance to health facilities was not a significant issue. At the community level, 68.7% lived in rural areas, and 30.5% were from the lake zone of mainland Tanzania (Table 2).

**Table 2:** Sociodemographic characteristics and distribution of women's autonomy in sexual and reproductive health among women in sexual union in Tanzania, 2022 demographic and health survey (*N* = 9,252**).**

Characteristics	*n* (%)	Women's autonomy in sexual and reproductive health, *n* (%)		*p*-value
		No	Yes	
Age category				<0.001
15–24	2,179 (23.5)	1,290 (59.2)	889 (40.8)	
25–34	3,511 (37.9)	1,687 (48.0)	1,824 (52.0)	
35–49	3,563 (38.6)	1,684 (47.3)	1,879 (52.7)	
Mean (±SD)	32.2 (8.7)			
Education level				<0.001
No formal education	1,887 (20.4)	1,292 (68.5)	595 (31.5)	
Primary	5,399 (58.3)	2,693 (49.9)	2,706 (50.1)	
Secondary/Higher	1,967 (21.3)	676 (34.4)	1,291 (65.6)	
Wealth index				<0.001
Poor	3,431 (37.1)	2,187 (63.7)	1,244 (36.3)	
Middle	1,761 (19.0)	909 (51.6)	852 (48.4)	
Rich	4,060 (43.9)	1,564 (38.5)	2,496 (61.5)	
Media exposure				<0.001
No	3,118 (33.7)	1,999 (64.1)	1,119 (35.9)	
Yes	6,134 (66.3)	2,661 (43.4)	3,473 (56.6)	
Working status				<0.001
Not working	2,953 (31.9)	1,732 (58.7)	1,221 (41.3)	
Working	6,299 (68.1)	2,928 (46.5)	3,371 (53.5)	
Parity				<0.001
None	604 (6.6)	375 (62.1)	229 (37.9)	
1–2	3,140 (33.9)	1,463 (46.6)	1,677 (53.4)	
3+	5,509 (59.5)	2,823 (51.2)	2,686 (48.8)	
Sex of household head				0.146
Male	7,964 (86.1)	4,043 (50.8)	3,920 (49.2)	
Female	1,288 (13.9)	617 (47.9)	672 (52.1)	
Distance to the health facility				<0.001
Big problem	2,919 (31.6)	1,684 (57.7)	1,235 (42.3)	
Not a big problem	6,333 (68.4)	2,977 (47.0)	3,356 (53.0)	
Husband/partner's age				<0.001
15–24	597 (6.5)	383 (64.2)	214 (35.8)	
25–34	3,024 (32.7)	1,547 (51.2)	1,477 (48.8)	
35–44	2,875 (31.1)	1,351 (47.0)	1,524 (53.0)	
45+	2,755 (29.7)	1,379 (50.1)	1,376 (49.9)	
Husband/partner's education				<0.001
No formal education	1,202 (13.0)	814 (67.8)	387 (32.2)	
Primary	5,785 (62.5)	2,987 (51.6)	2,798 (48.4)	
Secondary/Higher	2,265 (24.5)	859 (37.9)	1,406 (62.1)	
Community media exposure				<0.001
Low	2,302 (24.9)	1,498 (65.1)	804 (34.9)	
High	6,950 (75.1)	3,162 (45.5)	3,788 (54.5)	
Community poverty				<0.001
Low	5,743 (62.1)	2,454 (42.7)	3,290 (57.3)	
High	3,509 (37.9)	2,207 (62.9)	1,302 (37.1)	
Place of residence				<0.001
Urban	2,894 (31.3)	1,050 (36.3)	1,844 (63.7)	
Rural	6,358 (68.7)	3,610 (56.8)	2,748 (43.2)	
Geographical zones				<0.001
Western	808 (8.7)	614 (75.9)	194 (24.1)	
Northern	1,058 (11.4)	502 (47.5)	556 (52.5)	
Central	948 (10.2)	381 (40.2)	567 (59.8)	
Southern	1,857 (20.1)	761 (40.9)	1,096 (59.1)	
Lake	2,775 (30.0)	1,568 (56.5)	1,207 (43.5)	
Eastern	1,519 (16.4)	677 (44.6)	842 (55.4)	
Zanzibar	288 (3.1)	158 (55.0)	130 (45.0)	

### Prevalence of women’s autonomy in sexual and reproductive health

The prevalence of women's autonomy in sexual and reproductive health was 49.6% (95%CI: 47.5–51.8). Bivariate analysis revealed significant determinants of women's autonomy in sexual and reproductive health. Women aged 35–49 years (52.7%) had a significantly higher prevalence of autonomy in SRH than those aged 15–24 years (40.8%) (*p* < 0.001). Women with secondary education (65.6%) had a higher proportion of autonomy in SRH compared to women with no formal education (31.5%). Regarding socioeconomic status, women in the rich quantile had a significantly higher proportion of autonomy in SRH than their poor counterparts (61.5% vs. 36.3%). Women whose partner had attained secondary/higher education (62.1%) had a higher proportion of autonomy in SHR than those women whose partner had no formal education (32.2%). Women in urban areas had a higher proportion of autonomy in SHR than their counterparts (63.7% vs. 43.2%) (Table 2).

### Determinants of women’s autonomy in sexual and reproductive health

A multilevel mixed-effect logistic regression model revealed various determinants of women's autonomy in SHR. From the final adjusted model (Model II), women aged 35–49 years had higher likelihood of autonomy in SRH compared to women aged 15–24 years (AOR = 1.33, 95%CI: 1.09–1.61). Compared to women with no formal education, women with primary education (AOR = 1.49, 95%CI: 1.31–1.70) and secondary or higher education (AOR = 2.16, 95%CI: 1.83–2.55) were more likely to have autonomy in SRH. In terms of socioeconomic status, women in rich households were 19% more likely to have autonomy in SRH than those in poor households (AOR = 1.19, 95%CI: 1.02-1.40). Similarly, women with media exposure had higher odds of autonomy in SRH than their counterparts (AOR = 1.49, 95%CI: 1.33–1.67). Women who were working had 1.61 times higher odd of autonomy in SRH compared to their counterparts (AOR = 1.61, 95%CI: 1.45–1.78). Women with 1-2 children (AOR = 1.45, 95%CI: 1.18–1.78) and those with more than two children (AOR = 1.33, 95%CI: 1.07–1.65) had higher odds of autonomy in SRH compared to nulliparous women. At the community level, women in rural settings (AOR = 0.73, 95%CI: 0.61–0.87) were less likely to have autonomy in SRH compared to women in urban settings. Other community variables were not statistically significant in the final model, this may reflect the lack of statistical significance may reflect limited variability of these factors within clusters, potential overlap with individual-level characteristics, or insufficient statistical power to detect small effects once other covariates are controlled for (Table 3).

**Table 3:** Multilevel mixed-effect logistic regression model for determinants of women's autonomy in sexual and reproductive health in Tanzania, 2,022 demographic and health survey (*N* = 9,252).

Characteristics	Model 0	Model I	Model II	Model III
		AOR (95%CI)	AOR (95%CI)	AOR (95%CI)
Individual-level factors				
Age category				
15–24		1.00		1.00
25–34		1.16 (0.99–1.35)		1.15 (0.97–1.34)
35–49		1.36 (1.12–1.65)*		1.33 (1.09–1.61)*
Education				
No formal education		1.00		1.00
Primary		1.52 (1.33–1.73)*		1.49 (1.31–1.70)*
Secondary		2.21 (1.87–2.62)*		2.16 (1.83–2.55)*
Wealth index				
Poor		1.00		1.00
Middle		1.19 (1.04–1.35)*		1.11 (0.96–1.28)
Rich		1.45 (1.26–1.67)*		1.19 (1.02–1.40)*
Working status				
Working		1.58 (1.43–1.75)*		1.61 (1.45–1.78)*
Not working		1.00		1.00
Parity				
None		1.00		1.00
1–2		1.46 (1.19–1.79)*		1.45 (1.18–1.78)*
3+		1.32 (1.07–1.64)*		1.33 (1.07–1.65)*
Media exposure				
No		1.00		1.00
Yes		1.53 (1.37–1.71)*		1.49 (1.33–1.67)*
Sex of household head				
Male		1.00		1.00
Female		1.11 (0.97–1.26)		1.09 (0.96–1.25)
Husband age group				
15–24		1.00		1.00
25–34		1.22 (0.97–1.52)		1.21 (0.96–1.51)
35–44		1.28 (0.99–1.64)		1.27 (0.99–1.62)
45+		1.19 (0.91–1.56)		1.19 (0.91–1.55)
Community-level factors				
Community literacy				
Low			1.00	1.00
High			1.48 (1.20–1.82)*	1.20 (0.97–1.47)
Community poverty				
Low			1.00	1.00
High			0.70 (0.58–0.85)*	0.91 (0.75–1.11)
Residence				
Urban			1.00	1.00
Rural			0.63 (0.53–0.75)*	0.73 (0.61–0.87)*
Random effects				
Variance (SE)	0.67 (0.06)	0.46 (0.05)	0.47 (0.04)	0.44 (0.05)
PCV (%)	–	31.3%	29.9%	34.3%
ICC (%)	16.8%	12.3%	12.5%	11.7%
MOR	1.13	1.09	1.10	1.10
Model fitness				
AIC	12,084.05	11,659.66	11,959.88	11,641.17
BIC	12,098.30	11,773.61	11,995.49	11,776.48
Deviance	12,080.05	11,627.66	11,949.88	11,603.17

* *p* < 0.05, SE, standard error; PCV, proportional change in variance; MOR, median odds ratio; AIC, akaike information criterion; BIC, Bayesian information criterion.

### Measure of variations and model fitness

Table 3 also presents random effects and model fitness. Model 0 revealed a variance of 0.67 and a *p*-value of <0.001, suggesting a significant difference in women's autonomy in SRH. In terms of ICC, 16.8% of the variability of women's autonomy was attributed to differences between clusters, while 83.2% was due to individual-level differences within those communities. In Model I, the likelihood of women's autonomy in SHR was 1.36 times greater than for women with no autonomy. The best-fitting model was Model III, which had the lowest deviance (11,603.17) and Akaike Information Criterion (AIC) (11,641.17), as shown in Table 3.

## Discussion

This study aimed to assess the individual and community-level determinants of women's autonomy in SRH in Tanzania. The finding that 49.6% of women have autonomy in SRH is low, particularly in light of SDGs 3.7 and 5, which emphasize universal access to SRH services and women's empowerment. This highlights a need to strengthen efforts aimed at empowering women to make informed decisions regarding SRH, which is fundamental in improving maternal and child health outcomes. Our finding is lower than those reported in LMICs (26, 27), suggesting unique challenges within the Tanzanian context that necessitate deeper investigation. Conversely, our finding was higher than that reported in Nepal (19), indicating the complex and varied level of women's autonomy in SRH globally. This level of SRH autonomy among women in Tanzania, underscores how deeply entrenched gendered power imbalances, often perpetuated by patriarchal structures (28), limit women's agency and decision-making capabilities in critical aspects of their lives.

This study revealed that older women (35–49 years) were more likely to have autonomy in SRH. Our finding corroborates with previous studies in other parts of the world (19, 29). Age if often highlighted in the literature as factors that may influence women's ability to make informed decisions regarding SRH (29). This may reflect differences that emerge over time, such as increased exposure to information or gradual changes in social and economic roles (18). Contrarily, younger women may still be under the control of their in-laws or spouses in terms of making decisions. Therefore, they may have little to no autonomy in making SRH decisions (27). The contrasting dynamics underscore the importance of age-specific interventions to promote SRH autonomy.

Educated women were more likely to have autonomy in SRH compared to their uneducated counterparts in agreement with prior findings (19, 29). Education empowers women to have confidence, health literacy, negotiation skills and control over their SRH and reduces gender inequality (30). Additionally, educated women may acquire knowledge through employment and independent income. These compelling evidences explain why education is strongly associated with greater SRH autonomy, reinforcing the importance of investments in girls’ and women's education as part of efforts to improve reproductive health outcomes. Women in the rich quantile had twice the odds of exhibiting autonomy in SRH. This association may be related to the difference in economic resources, which can enhance access to information and healthcare services and may be linked with greater participation in household decision-making (31, 32).

Women with one or more children showed higher odds of autonomy in SRH compared with nulliparous women. This finding suggests that childbearing may enhance women's confidence and capacity in decisions-making to matters related to SRH. Our finding is corroborates to a multicounty study in SSA (33). Parenthood often brings increased interaction with the health system, exposure to reproductive health services, and greater familiarity with contraceptive options, which may contribute to improved autonomy. In most settings, women who have begun childbearing may also experience increased social recognition within their households and communities, which can translate into stronger influence over reproductive decisions.

Our study found a disparity between the individual education and the lack of a significant effect from community literacy. This can be clarified by considering the mechanisms through which each variable operates. Individual education directly empowers women by enhancing their knowledge, critical thinking skills, economic opportunities, and self-efficacy (34). This education thereby increasing their autonomy to negotiate for reproductive health autonomy within their relationships. Conversely, community literacy may not directly translate into challenging deeply entrenched gender inequities, or power imbalances at the household level (35, 36). A literate community might still uphold traditional patriarchal structures (36). This might hold individual women, find their decision-making constrained by prevailing social expectations despite their literacy.

Women with media exposure had higher likelihood of having autonomy in SRH than their counterparts (31, 37). Exposure to television, radio, newspapers, and other media platforms empowers women to exercise autonomy in SRH. This finding underscores the need to use media channels strategically to leverage information regarding Sexual and Health Reproductive Health Rights. We also found that working women were more likely to have autonomy in SRH compared to non-working women. Working women may have financial independence and decision-making power (38, 39). Financial gain may promote women's capacity to negotiate for her reproductive rights and access services without relying entirely to spouse or family members.

Women residing in rural areas were significantly less likely to have autonomy in SRH decisions compared to their urban counterparts (7, 40). This notable disparity may be influenced by limited access to health facilities, lower levels of education, traditional gender norms, and reduced exposure to SRH information in rural settings (41, 42). These findings highlight the need for targeted interventions in rural communities. Such efforts could focus on empowering women, enhancing their access to quality SRH services, and ensuring the widespread dissemination of accurate SRH information to bridge this significant urban-rural gap and promote equitable access to reproductive rights.

## Strengths and limitations

This study utilizes a large sample size drawn from nationally representative data collected with an experienced field assistant. The use of multilevel analysis, appropriate for the hierarchical structure of the DHS data, ensures that standard errors and estimates are reliable. However, the study also has some limitations. Since the DHS survey relies on self–reports and involves sensitive questions, there may be potential recall and social desirability biases. Cross-sectional designs inherently limit causal inference by capturing data at a single point in time, preventing the establishment of temporal relationships between variables. Additionally, the complexity of autonomy measures can introduce subjectivity and potential biases, thus weakening the strength and generalizability of the study's conclusions. This study as well encountered limitations in generalizability, as only women in sexual relationships were included. The findings for this study should cautiously interpreted based on the above limitation.

## Conclusion

This study has revealed that nearly half of the women lacked autonomy in SRH. The 49.6% indicates that nearly half of the women are left behind regarding SDGs 3.7 and 5. The findings demonstrate that both individual and community-level factors significantly influence the uptake of women's autonomy towards SRH. At the individual level, older women's age (35–49 years), higher education, living in a wealthy household, and media exposure are positively associated with women's autonomy in SRH. At the community level, women in rural areas were significantly less likely to have autonomy in SRH. The study's findings align with Connell's emphasis on the dynamic interplay between individual experiences and broader social structures. This suggests that while individual empowerment is vital, systemic changes are also necessary to dismantle the patriarchal norms that underpin limited SRH autonomy, requiring a multi-faceted approach that considers both individual agency and the broader gender order.

Furthermore, there is a compelling need for robust, age-specific programs that empower younger women to make independent SRH choices, potentially by integrating comprehensive SRH education into secondary school curricula and community-based youth programs. The findings of this study show that media platforms like television, radio, and digital media can be instrumental in disseminating information about SRH rights and services, thereby increasing awareness and challenging traditional norms that hinder women's autonomy. Additionally, socio-economic empowerment initiatives, such as financial literacy programs and support for women entrepreneurs, should be expanded, especially in rural areas, to enhance women's negotiating power within their households and communities. Therefore, these efforts when they are combined with improved access to quality healthcare services in underserved regions, will be crucial in aligning Tanzania with Sustainable Development Goals 3.7 and 5, ultimately improving maternal and child health outcomes.

## Data Availability

Publicly available datasets were analyzed in this study. This data can be found here: https://dhsprogram.com.
